# A Novel, Multifunctional, Floatable, Lightweight Cement Composite: Development and Properties

**DOI:** 10.3390/ma11102043

**Published:** 2018-10-19

**Authors:** Zhenyu Huang, Fang Wang, Yingwu Zhou, Lili Sui, Padmaja Krishnan, Jat-Yuen. Richard Liew

**Affiliations:** 1 Guangdong Provincial Key Laboratory of Durability of Marine Civil Engineering, Shenzhen University, Shenzhen 518060, China; wwybest@163.com (F.W.); ywzhou@szu.edu.cn (Y.Z.); 2 Department of Civil and Environmental Engineering, National University of Singapore, Singapore 117576, Singapore; ceekp@nus.edu.sg (P.K.); ceeljy@nus.edu.sg (J.-Y.R.L.)

**Keywords:** compressive strength, cenospheres, glass microspheres, fiber-reinforced, floating concrete, lightweight concrete

## Abstract

This paper presents the development of a novel, multifunctional, floatable, lightweight cement composite (FLCC) using three different types of glass microspheres for structural engineering applications. Eight different mixtures of FLCC were produced and their matrix-related parameters were examined experimentally by adopting different types of microsphere fillers, fiber content (polyethylene fibers (PE)), and water-to-binder ratios. Along with the mechanical properties such as compressive, flexural, tensile strengths, and modulus of elasticity, the water tightness of the material was evaluated by sorptivity measurements and the energy efficiency by thermal conductivity. The optimal FLCC has an oven-dry density of 750 kg/m^3^, compressive strength (**f_cm_**) up to 41 MPa after 28-day moist curing, low thermal conductivity of 0.152 W/mK, and very low sorptivity. It is found that an optimized amount of PE fiber is beneficial for improving the tensile resistance and ductility of FLCC while a relatively large amount of microspheres can increase the entrapped air voids in the FLCC matrix and reduce its density and thermal conductivity. Microstructural analysis by scanning electron microscopy (SEM) reveals that the microspheres are distributed uniformly in the cement matrix and are subjected to triaxial compression confinement, which leads to high strength of FLCC. Segregation due to density difference of FLCC ingredients is not observed with up to 60% (by weight) of glass microspheres added. Compared to the other lightweight aggregate concretes, the proposed FLCC could be used to build floating concrete structures, insulating elements, or even load-bearing structural elements such as floor and wall panels in which self-weight is a main concern.

## 1. Introduction

In the past decade, the construction industry all over the world has been exploring solutions to improve the productivity in site construction. The focus was placed on the reduction of site manpower usage at reasonable cost while maintaining design variety and high-quality work. Concrete is one of the most widely used materials in construction. Nevertheless, lately the focus has shifted to sustainable development and various industries are striving to save energy and lower the environmental impact. Concrete is more frequently required to possess more advanced characteristics to achieve low density, low cost, better thermal insulation, superior mechanical properties, and eco-friendliness. As a result, a multifunctional, lightweight, high-strength concrete is a superior alternative material used for structural elements such as building floors, exterior wall panels, and roofing tiles in which self-weight needs to be reduced significantly. The main advantage of using lightweight concrete is the reduction of self-weight of structural members and overall structures, allowing smaller sizes of structural members and less reinforcing materials. Thus, requirements for foundations and transportation cost can be reduced significantly. Additionally, it allows for easy and rapid installation at construction sites due to its lower self-weight, thereby saving time and improving productivity in construction.

**Figure 1:**
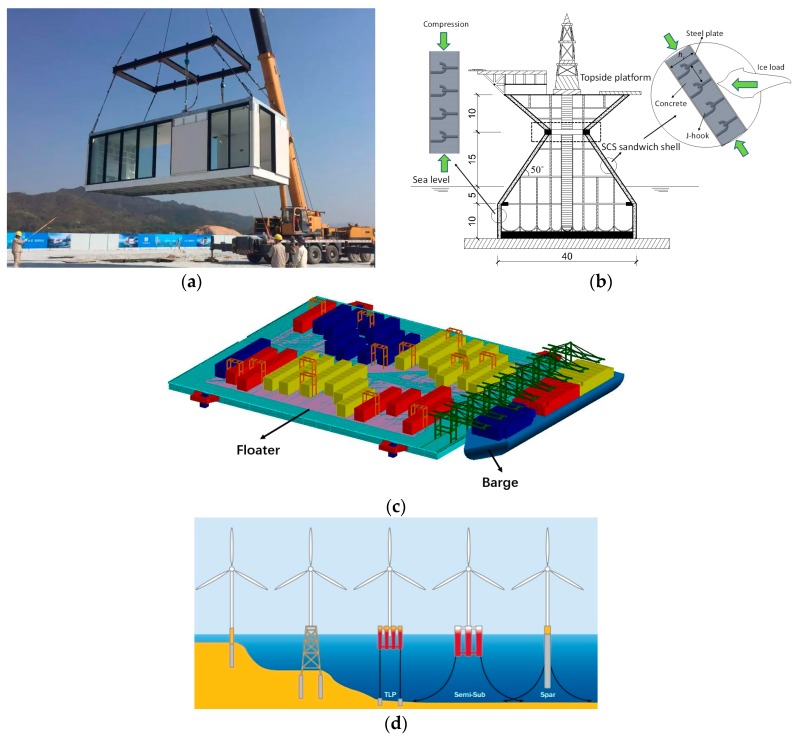
Onshore and offshore constructions using lightweight concrete: (**a**) prefabricated modular construction; (**b**) offshore platform [3]; (**c**) offshore floater; (**d**) offshore wind farm [4,5].

Lightweight onshore and offshore constructions shown in Figure 1a–d have become more and more promising in recent years. Lightweight concrete is attractive in structural components such as floor and wall panels, whose weight make up around 60% of the total self-weight of modular units [1,2]. Using lightweight concrete, the overall self-weight of the constructed structures could be much lighter than conventional concrete structures. For offshore floating structures, the platform is light enough to float and can be launched and ballasted so that it floats upright [3,4,5]. It will then be wet towed to the site for further assembly. It is essential that during the process of wet towing, the lightweight units are not compromised and their structural capacity is preserved.

Definitions of lightweight concrete vary among international standards and codes of practices. Table 1 lists the technical specifications of lightweight concrete for international codes, including CEB-FIP [6], ACI 213R [7], ASTM C330 [8], BS EN 13055 [9], and Chinese JGJ 51 [10]. In general, the unit weight of lightweight concrete ranges from 800 to 1950 kg/m^3^ while for structural lightweight concrete, ACI 213 recommends the range as 1120 to 1920 kg/m^3^ with compressive strength larger than 21MPa [7]. The methods for production of lightweight concrete can be mainly summarized as follows: (1) Adding lightweight aggregates into concrete mixtures, replacing completely or partially normal weight aggregates to achieve lightweight property. Conventional lightweight aggregates concrete incorporating expanded clay, expanded glass, expanded shale, expanded perlite, expanded vermiculite, foamed slag, expanded polystyrene beads, and fly ash aggregates has been developed and investigated in the past and has successfully achieved desired strength and unit weight for application [11,12,13,14,15,16]. (2) Adding foaming agent or air-entraining agent into the mixtures to introduce a certain volume of air voids in concrete mixtures [17,18]. However, this method could reduce the mechanical properties of concrete even though it could lower the density and increase the workability. (3) Gap grading of mixtures (sand is eliminated and voids are between coarse aggregate particles), for example, pervious concrete for pavement [19].

Extensive research has been done on the development of lightweight concrete. Lightweight concrete is generally weaker than normal concrete due to the brittleness and low modulus of lightweight aggregates, which to some extent restricts its wide application. Experimental investigations by Neville [20] showed that both mechanical properties and thermal conductivity of lightweight concrete show significant relation to density. There was a linear correlation between the thermal conductivity and the density of lightweight concrete (LWC) produced with different types of lightweight aggregates (LWAs), for example, pumice, vermiculite, cinders, expanded shale, and expanded slag. Chandra and Berntsson [21] presented a relationship between the compressive strength and density of LWC, applying expanded clay as LWA, for which the compressive strength of LWC increased from 7 to 16 MPa with density varied from 1000 to 1500 kg/m^3^. For structural application, Mydin et al. [22] and Prabha et al. [23] investigated the compression performance of a load-bearing wall system with lightweight foamed concrete infilled in double-skin profiled steel sheeting. However, the compressive strength of the foamed concrete was so low (around 6–8 MPa) that it had limited application in building construction (e.g., low-rise temporary housing). Additionally, particle characteristics of lightweight aggregates (LWAs) have great influence on the mechanical performance of the concrete [24]. To improve the ductility of LWC, fibers may be added to LWC [25]. Three types of fibers, metallic fibers, polymer fibers, and natural fibers, are utilized widely in concrete. While the density and thermal conductivity would be increased due to the higher density and thermal conductivity of metallic fibers, polymer fibers could be a good alternative to further improve both mechanical and functional properties without affecting the density and thermal conductivity.

Recent development on ultralightweight cement composite (ULCC) by Wu et al. [26], Huang et al. [27,28,29,30,31,32] showed that ULCC using fly ash cenospheres exhibited low density ranging from 1250 to 1550 kg/m^3^, high compressive strength up to 87.3 MPa, high flexural strength of 11.4 MPa, and deflection hardening behavior by using low steel fiber content (0.5% in volume) [33], which has been applied in steel–concrete–steel sandwich composite beams [27,30], walls [4,29,31], and shells [27,28,32] within marine offshore areas. As can be seen, use of lightweight fly ash cenospheres in cement-based composites has been an area of interest and a growing number of researchers investigate its mechanical and functional properties [33,34,35,36,37,38,39,40] and promote its application subjected to different loading scenarios [27,28,29,30,31,32,41]. Cenospheres are lightweight (400–800 kg/m^3^), high-strength (crushing strength up to 45 MPa), inert, hollow spheres made largely of silica and alumina and filled with air or inert gas, typically produced as a by-product of coal combustion at thermal power plants or artificial sintering method. Therefore, the resulting concrete using cenospheres exhibits high strength and lightweight behavior. Compared to conventional lightweight aggregates, cenospheres have lower water absorption due to their thick shell and higher crushing strength with very light unit weight. While the cenospheres provide an excellent choice for such lightweight cement composites (LCC), an important question remains on how the crushing strength of these lightweight microspheres affects the strength of the LCC. Would the strength of the LCC be higher if the crushing strength of microspheres can be increased? Artificial fine microspheres aggregates, namely, soda–lime–borosilicate-based glass microspheres, were adopted as an alternative to FAC to improve the mechanical and even functional properties of lightweight concrete [42,43,44,45]. The use of glass microsphere (GM) products has increased tremendously in recent years [46,47,48,49,50,51]. It was estimated that high-performance glass microspheres were another type of ultralightweight, inorganic, nonmetallic material with a hollow structure, and they were a versatile and high-performance new filler developed. They owned features with light weight, large bulk, low thermal conductivity, high crushing strength, and good flow properties, which would be an excellent filler material for cement composites. However, the past research revealed that the cement composites incorporating glass microspheres had low compressive strength and elastic modulus [39,40,41,42,43,44,45,46,47,48,49,50,51]. 

Table 2 provides a summary on the properties of lightweight cementitious materials using FAC and GM available in recent published data. Interestingly, the presence of fly ash is known to reinforce the structure of the composite by improving the interfacial transition zone and leads to better fracture toughness and compressive strengths [52,53]. Although there is extensive functional ultralightweight concrete with good thermal insulation properties, the compressive strength and elastic modulus is too low for application in structural elements. Cracks may occur during transportation and lifting of such elements using glass-microspheres-based lightweight concrete. Thus, the concrete durability life may be significantly reduced. Therefore, there is an essential need to develop a functional structural material with excellent mechanical properties, low thermal conductivity, and low permeability to resist ingress of water.

This paper presents the research and development work on a floatable, lightweight cement composite (FLCC) using glass microspheres and investigates its mechanical and thermal properties, resistance to water penetration, and microstructure. Effects of different types of glass microsphere aggregate, water-binder ratio, and fiber content on compressive strength, flexural strength, tensile strength, ductility, elastic modulus, sorptivity, workability, and density of FLCC were evaluated experimentally. Potential challenges of FLCC for structural application are also discussed. This study would be useful for design and application of prefabricated and precast structural members such as partition wall panels, flooring slabs, ceilings, and insulation boards for onshore and offshore infrastructures.

## 2. Materials and Mixing Methodology

### 2.1. Materials

Ordinary Portland Cement with specific gravity of 3.16 (ASTM Type I cement) was used as the binder material in the FLCC mixtures. Lightweight, high-strength glass microspheres (GM) and fly ash cenospheres (FAC) were used as microaggregates for the FLCC. Densified Elkem Microsilica 940U silica fume (SF) with SiO_2_ content of over 90% was used in all mixtures to strengthen the bond strength of the interface transition zone (ITZ) between the microspheres and cement paste. To gain a proper workability, a high range of polycarboxylate-based superplasticizer was used to achieve required flow around 200 mm based on flow table test [55]. The chemical composition of OPC, SF, FAC, and GM are presented in Table 3. Glass microspheres used in the FLCC had an average particle density of approximately 380–630 kg/m^3^ with relative high crushing strength from 27.6 to 68.9 MPa, while FAC had average bulk density of 450kg/m^3^ with relative high crushing strength of 15.0 MPa, as shown in Table 4. The crushing strength of the aggregates is directly correlated to the particle density. 

**Figure 2:**
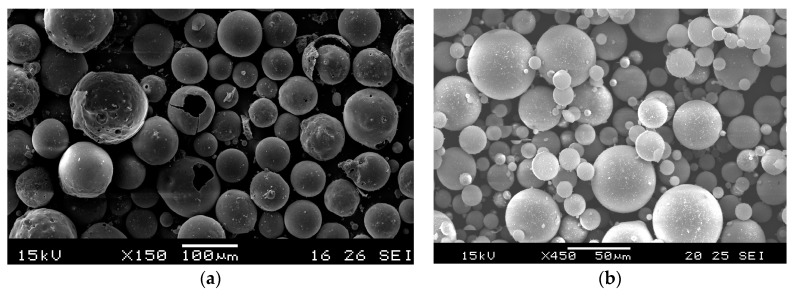
SEM image of lightweight fillers: (**a**) fly ash cenospheres; (**b**) glass microspheres (GM46).

**Figure 3:**
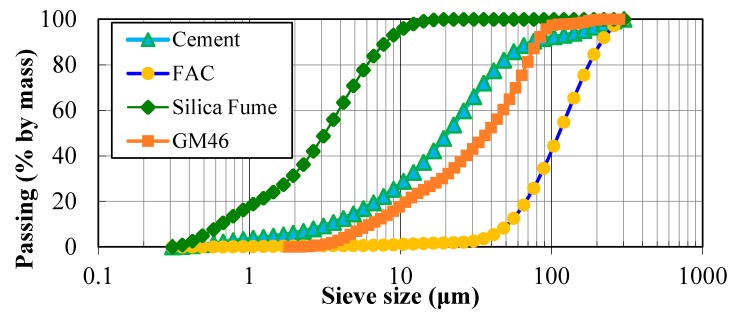
Particle distribution of raw materials.

The used FAC and GM had a relatively smooth surface and were composed of a closed external shell and internal air pores encapsulated within the shell. This could be the reason that the GM and FAC had lower water absorption (less than 2% after soaking in water for 1 h) compared to the other available lightweight aggregates (LWAs). Glass microspheres used in this paper had a diameter in the range of 2–125 microns with wall thicknesses less than 1 micron. Figure 2a,b shows the SEM image of two types of cenospheres (FAC and GM) and both aggregates are almost intact and have good spherical shape. Particle size distribution of these two materials is illustrated in Figure 3. Glass microspheres had fineness comparable to that of cement with mean particle size of 40 μm.

To improve the flexural strength and ductility of FLCC, different amounts in volume of polyethylene (PE) fibers were employed. PE fiber has a tensile strength around 2900 MPa with an elastic modulus of 116 GPa. The aspect ratio of PE fiber (fiber length to diameter ratio) is 720. According to the information from the manufacturer, the PE fibers were not surface treated. A total of three mixtures were added with PE fiber ranging from 0.5% to 1.5% in an interval by volume. 

### 2.2. Mix Proportion

Floatable, lightweight cement composite was cast using above-mentioned OPC, superplasticizer, silica fume, and glass microspheres with a water/binder ratio of 0.35 to 0.65. Due to the fine particle size and corresponding larger specific surface area of the glass microspheres, more water should be used, hence a w/b of 0.6 was chosen based on trial tests. The density of lightweight fine aggregate affects the density of FLCC considerably, since the volume of aggregate is significant in the mixtures. Three types of glass microsphere aggregates with a mass ratio of 0.6 were considered in the mix proportions. Another two control mixes were ULCC using fly ash cenospheres and LWC using expanded shale aggregates. The mix proportions of FLCC, ULCC, and LWC are summarized in Table 5. Trial batches were made to obtain the desired density, slump flow, and strength, therefore the contents of superplasticizer and fine aggregates were adjusted.

### 2.3. Mixing Procedures

Unlike normal concrete, making FLCC requires longer mixing time due to the fine nature of the microspheres, while the spherical shape provides a ball-bearing effect and ensures better flowability of the FLCC. For workability measurement, the fresh FLCC mixture was placed in a truncated conical mold on a plastic board [55]. The FLCC spread out on the board after lifting the mold vertically. Time for the FLCC to reach a diameter of 200 mm and final average spread diameter in two directions were measured for evaluation of the workability. Due to the ball-bearing effect of glass microspheres, the successful FLCC mixture is a highly workable composite similar to the cement grout which is suitable for pumping and grouting in construction. It should be noted that the quality control of producing the FLCC needs to be considered carefully. A high-shear mixer should be adopted to improve the distribution of fibers when mixing FLCC. Due to the use of microaggregates, FLCC can be adopted for advanced extrusion methods and 3D printing technology, provided that adequate quality control exists. Then, less porosity and denser microstructure can be achieved, leading to better mechanical properties.

### 2.4. Test Methods

Table 6 lists the test method references and specimen size for measuring the mechanical and functional properties of FLCC. The workability was performed immediately after production of FLCC according to British Standard EN 1015-3 [55]. The fresh density and air content were measured in general compliance with the standard procedures in ASTM C138 [56]. The 100 mm diameter, 200 mm long cylindrical specimens, and 50 mm cube specimens were used to determine the density in hardened concrete. The compressive strength, modulus of elasticity, and Poisson’s ratio of the cylindrical specimens were measured according to ASTM C39 [57], ASTM C109 [58], and ASTM C 469 [59], respectively. The modulus of elastic values of FLCC specimens was also calculated by Equation (1) to (2), given by ACI 318 [60] and CEB FIP code [6], respectively.

(1) $$E_{\mathrm{ACI}}=0.043w^{1.5}\sqrt{\sigma }$$

(2) $$E_{\mathrm{CEB}-\mathrm{FIP}}=0.017w^{2}\sigma^{0.33}$$

where E = modulus of elasticity, w = air dry density of concrete, σ = compressive strength.

The flexural strength of FLCC was measured using three-point bending on 40 × 40 × 160 mm prism specimens according to BS EN 196-1 [61]. Additionally, the compressive strength of FLCC can be calibrated using portions of prisms broken in flexure following ASTM C349 [62]. To demonstrate the feasibility of using FLCC as a lightweight engineering cement composite (ECC) with strain-hardening properties, direct tensile tests on FLCC incorporating PE fibers were also conducted using three dogbone-shaped specimens recommended by the Japan Society of Civil Engineers (JSCE) [63] for standardized testing of ECC at the age of 28 days.

The thermal conductivity of the FLCC and normal concrete specimens was determined according to ASTM C518 [64] by a heat flow meter and ASTM C177 [65] using guarded hot plate equipment. The results reported in this paper were the average value from two 300 mm × 300 mm × 50 mm plate specimens. The 300 mm × 300 mm × 50 mm plate specimens used were first moist cured for 28 days, and then dried in a laboratory environment for two weeks followed by drying in an oven at 105 °C until constant weight was obtained. For the tests in this paper, about two months were needed to achieve a constant weight.

**Figure 4:**
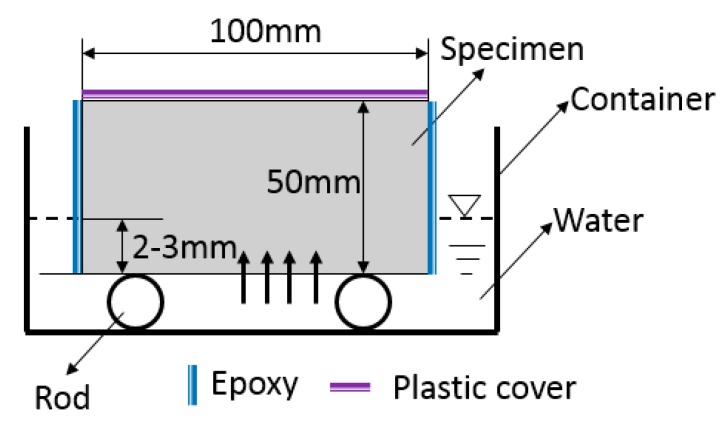
Schematic diagram of sorptivity test.

The sorptivity test is a useful test to examine the effectiveness of any water-repellent materials. Sorptivity of FLCC, ULCC, NWC, and LWC was determined by measuring the weight increase of a specimen due to the absorption of water as a function of time when one surface of the specimen was exposed to water according to ASTM C 1585 [66]. Figure 4 shows the test setup for the FLCC sorptivity test. Both ends of the specimens were well grinded. The specimens were placed in an environmental chamber at a temperature of 50 °C and relative humidity of 80% for 3 days before being stored in a sealable plastic bag at 23 ± 2 °C for 15 days. The specimens were coated with epoxy on side surfaces to allow only one surface of the specimen to be in contact with water, with a depth of water between 2 and 3 mm (Figure 4). The top surface of the specimens was covered to prevent evaporation during the test. The bottom surface was in contact with water and the weight increase of the specimen was monitored at times. The test consisted of registering the increase at given intervals of time (1, 2, 3, 4, 6, and 24 h) when permitted to absorb water by capillary suction. According to ASTM C 1585, two parameters, (1) initial water absorption, namely, the quantity of water absorbed by a unit surface area during the first hour of the test, and (2) sorptivity parameter, namely, the slope of the linear regression curve of the quantity of water absorbed by a unit surface area versus square root of the elapsed time from 1 to 24 h, were calculated.

## 3. Experimental Results

### 3.1. Density, Workability, Air Content, and Compressive Strength

**Figure 5:**
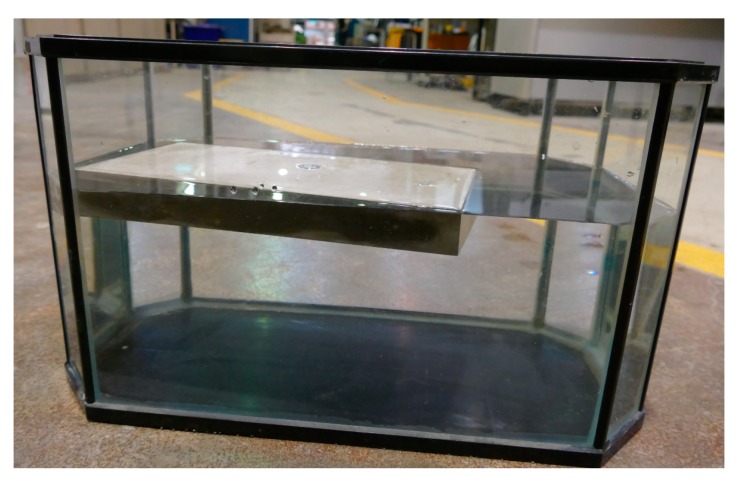
FLCC specimen floating in a water tank.

Table 7 summarizes the measured properties of FLCC, including flow value, density, 28-day compressive strength, and flexural strength. The average fresh density of FLCC obtained ranges of 880–970 kg/m^3^. During the mixing, it was found that a higher amount of superplasticizer was needed, which was due to the larger specific surface area of lightweight glass microspheres compared to the normal angular particles of crushed stone aggregates. Increasing the amount of the glass microspheres resulted in a corresponding decrease in the compressive strength and density. Different amounts of PE fiber addition resulted in different increases of flexural strength but in certain reductions of compressive strength. The 28-day compressive strength of FLCC ranged from 26.9 MPa to 41.0 MPa. This is a promising property that indicates FLCC could be used as a structural cement composite material in terms of compressive strength. The high strength of FLCC may be due to: (1) the high strength of the fine glass microsphere aggregate itself, and (2) the three-dimensional confinement effect from the surrounding hardened cement matrix when subjected to loading, which is attributed to uniform distribution of glass microspheres in the cement matrix. Among those mixtures, FL40 had the highest compressive strength of up to 41 Mpa, followed by FL46 and FL60 with slightly lower compressive strengths. Interestingly, it was found that while the decrease in compressive strength from FL40 to FL46 corresponded to the reduction in density, the decrease in compressive strength from FL46 to FL60 was much higher, indicating that a threshold may exist in terms of the type and density of GM that can be added to cement composites. Therefore, FL46 was chosen as the optimal type of GM for other mixes, including those containing PE fibers. For a given w/b ratio of 0.6, the FL60 sample had a higher porosity in the cement paste matrix but lower porosity in the microspheres compared with FL40 and FL46. Compared to a w/b ratio of 0.35, FL46-35 had a higher porosity of 7.51%, which may be due to the low w/b ratio. The densities of all FLCC were less than the density of water, which indicated that the FLCC concrete can float on the water. Figure 5 shows a floating sample of FLCC when it was placed in water. A more promising behavior was that a broken FLCC segment could also float on water, indicating that glass microsphere aggregates were uniformly distributed in the cement matrix. This suggests that even if failure of a structure using FLCC occurs, it would not sink into the water.

**Figure 6:**
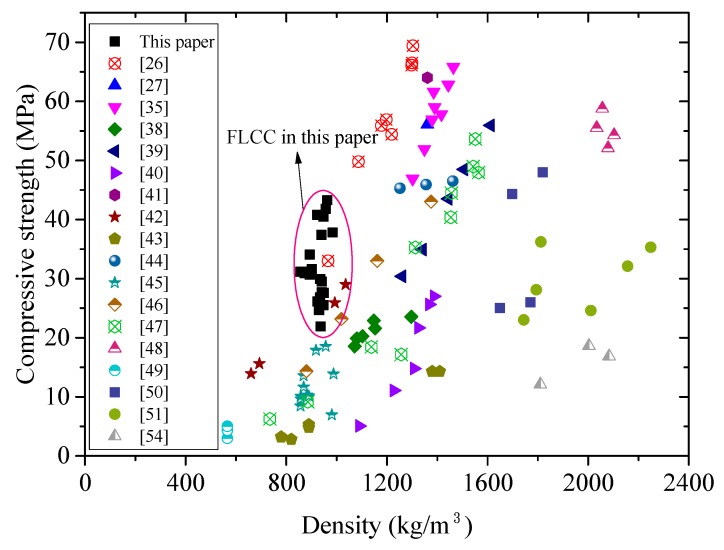
Relationship between compressive strength (**f_cm_**) and density of lightweight concrete using cenospheres and glass microspheres.

Figure 6 shows the database of lightweight concrete using fly ash cenospheres and glass microspheres in terms of density and compressive strength (**f_cm_**). Both the test data in this paper and those from literature are plotted in the figure. Compared to other types of lightweight concrete, FLCC developed in this paper exhibited much higher specific strength (compressive strength-to-density ratio was from 28.6 to 42.3 kPa/kg·m^−3^, while for S355 steel it was 45.2 kPa/kg·m^−3^). Also, among the test data with density below 1000kg/m^3^, FLCC showed promising performance with compressive strength (**f_cm_**) exceeding 40 MPa. For concrete with density grade between 1000 kg/m^3^ and 1300 kg/m^3^, FAC-based ULCC exhibited the highest specific strength up to 53.3 kPa/kg·m^−3^ [26,35].

### 3.2. Flexural and Tensile Strength

**Figure 7:**
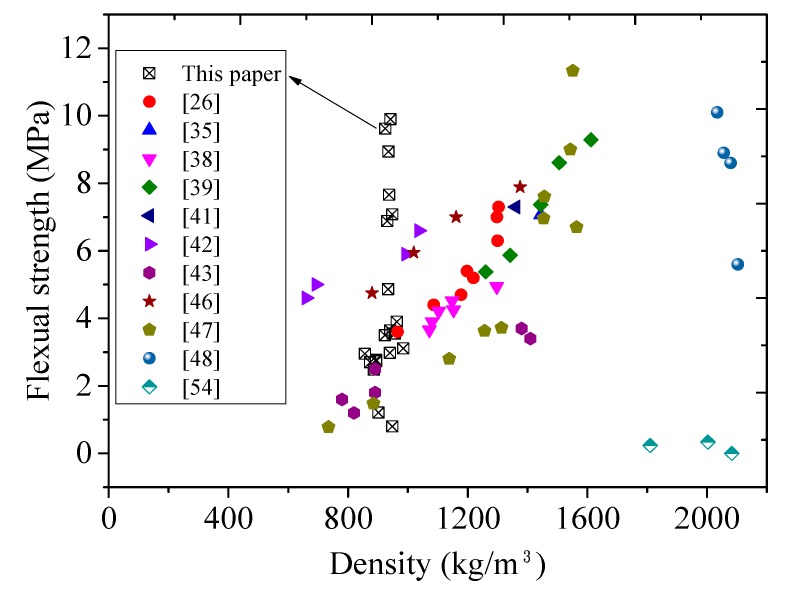
Relationship between flexural strength (f_ct_) and density of lightweight concrete using cenospheres and glass microspheres.

**Figure 8:**
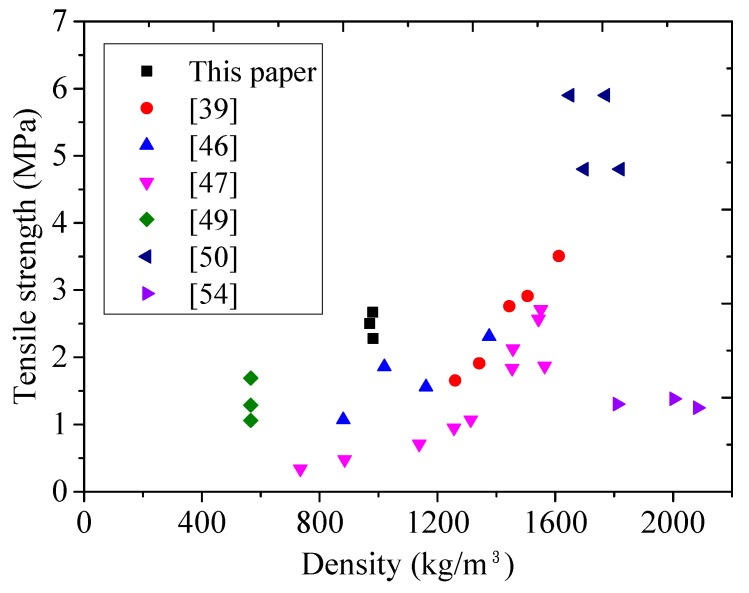
Relationship between tensile strength and density of lightweight concrete using cenospheres and glass microspheres.

With the addition of 0.5%, 1.0%, and 1.5% PE fiber, the flexural strength (f_ct_) increased by about 80.3%, 223.3%, and 325.6%, respectively, compared to that without fiber addition. The average 28-day flexural strength through three-point bending test ranged from 4.0 MPa to 9.5 MPa. Figure 7 and Figure 8 plot the relationship between flexural/tensile strength and density of published lightweight concrete using FAC or GM, respectively, showing that FLCC possessed the highest flexural/tensile strength for 1000 kg/m^3^ grade ultralightweight cement composite. 

**Figure 9:**
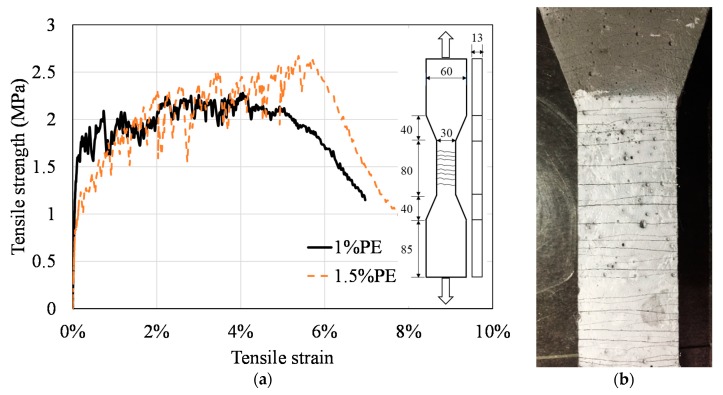
Tensile behavior of FLCC: (**a**) stress–strain curves of FL46; (**b**) multiple-cracking behavior.

To check if the FLCC can be developed as a lightweight cement composite with strain-hardening properties, direct tensile tests on FLCC incorporating PE fibers were conducted using three dogbone specimens at the age of 28 days. The representative stress–strain curves of FLCC with 1% and 1.5% PE fibers are shown in Figure 9a. It was found that the direct tensile strain capacity of these two mixtures exhibited strain-hardening behavior under direct tensile load. The maximum tensile strains both exceeded 3%, up to about 5–6%, which is higher than that of normal ECC [67]. Multiple cracks were observed, initiating and propagating all over the specimen surface. Figure 9b also shows the residual crack pattern of FLCC after the tensile test. These behaviors suggest that the developed FLCC incorporated with a low amount of PE fibers (less than 2%) is qualified for the development of high-ductility ECC. The glass-microsphere-based cement matrix satisfies the micromechanics-based design criteria for strain-hardening cement composites which are: (1) the tensile first-cracking strength must not exceed the maximum fiber-bridging strength, and (2) the maximum energy available for steady-state flat crack propagation much exceed the energy required for matrix breakdown [68]. Potentially, FLCC with PE fiber used in this paper could be developed as a novel type of ultralightweight, strain-hardening cement composite. Nevertheless, this paper only handles the preliminary behavior of FLCC, while design method and performance evaluation on such ultralightweight, strain-hardening cement composites need further study.

### 3.3. Elastic Modulus

**Figure 10:**
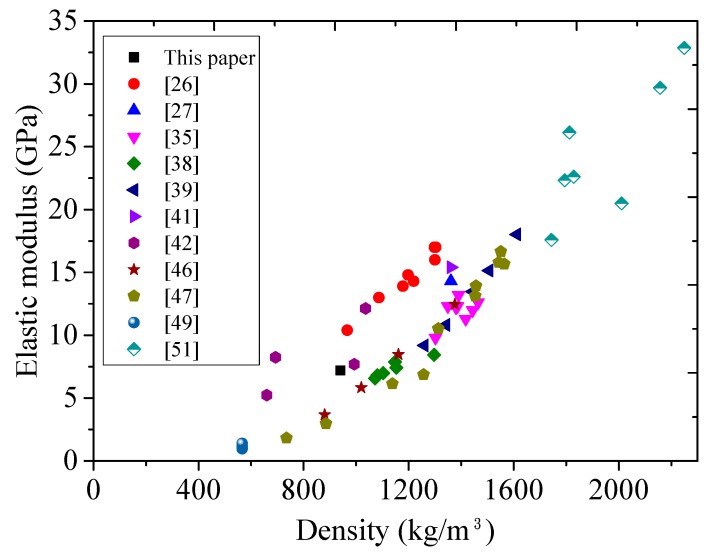
Relationship between elastic modulus and density of lightweight concrete using cenospheres and glass microspheres.

The modulus of elasticity and Poisson’s ratio of FLCC are 7.2 GPa and 0.25, respectively, based on ASTM C469. Figure 10 plots the relationship between elastic modulus and density of published lightweight concrete using cenospheres and glass microspheres. In general, the elastic modulus is proportional to the density of the FLCC. Compared to ULCC and normal LWC, FLCC has lower density and elastic modulus due to lower elastic modulus of glass microsphere aggregates, but with comparable compressive and flexural strength. This fact may challenge the serviceability design for flexural-dominated structural members in future applications. However, the drawback of low elastic modulus can be addressed by metal-FLCC composite design, which could be a reasonable engineering solution. 

### 3.4. Sorptivity

**Figure 11:**
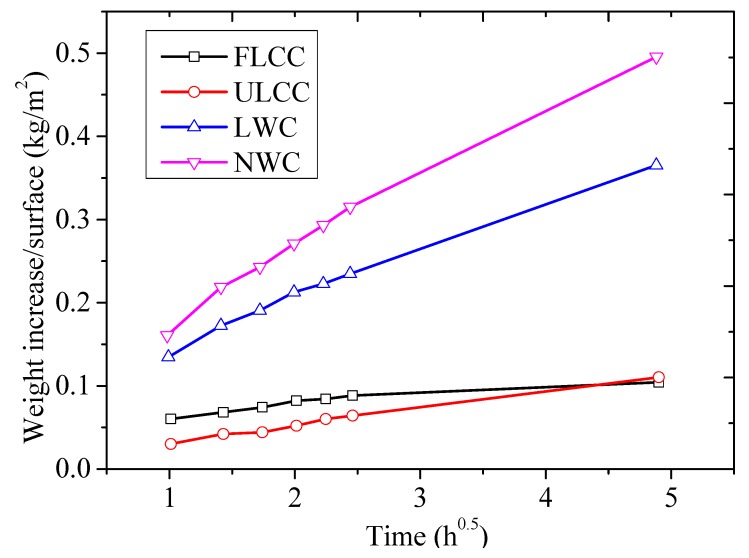
Average weight increase per area against time for different lightweight concretes.

In this paper, the mix FL46 was selected for the sorptivity test. Three Ф 100 mm × Ф 50 mm specimens were cut from a Ф 100 × Ф 200 mm cylinder after the 28-day moist curing. Figure 11 compares the average weight increase due to water absorption against time of different concretes, including FLCC, ULCC, LWC, and NWC. It was found that FLCC had lower water absorptivity compared to that of normal-weight concrete and lightweight aggregate concrete, and had similar water absorptivity with ULCC, which is essential to retain low unit weight if FLCC is exposed in a marine environment.

### 3.5. Thermal Conductivity

**Figure 12:**
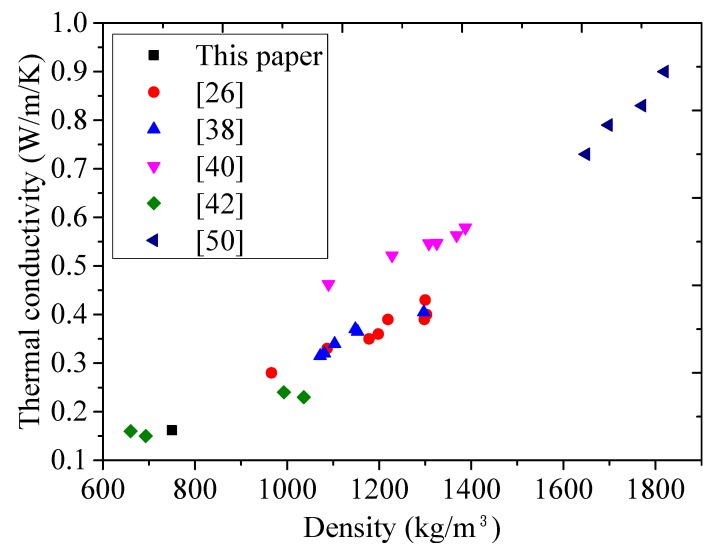
Relation between thermal conductivity and oven-density for different lightweight concretes.

Two FLCC specimens were prepared for thermal conductivity measurement. Figure 12 shows the relationship between thermal conductivity and density for lightweight concrete using FAC and glass microspheres. While the thermal conductivity is dependent on a number of factors, such as moisture content, type of aggregates, w/b ratio, and so forth, the most important parameter affecting the thermal conductivity is the density. Introduction of air voids lowers the density and decreases the thermal conductivity of concrete. The lower the density of lightweight concrete, the smaller the thermal conductivity when ignoring the moisture content. Glass microspheres have lighter weight and lower thermal conductivity (0.03–0.044 W/mK) than that of FAC (0.065–0.08 W/mK) and other denser aggregates (sand and granite: 2.6 W/mK). From Table 8, comparing ULCC-6 with w/b ratio of 0.56, and FL46 with w/b of 0.60, the difference in density is mainly due to the density of microspheres and therefore, the difference in the thermal conductivity may be attributed to the glass microspheres. The thermal conductivity of the FLCC (0.162 W/mK) at ambient temperature (30 °C) was 42% lower than that of the ULCC-6 (0.28 W/mK), while that of ULCC-1 was 80% lower than that of the normal concrete (1.98 W/mK) (Table 8). It is known that the heat transfer through opaque envelopes is directly related to its thermal conductivity. Therefore, adopting FLCC with low thermal conductivities can result in lower heat transmitted inside the building. This is especially beneficial in warm climates to reduce the energy incurred by air conditioning. As temperature increased, the thermal conductivity of FL46 increased from 0.154 to 0.162 and 0.180 W/mK, respectively, from 10 °C to 30 °C and 90 °C. But the increment was very small. Due to the limitation of the test facility in this paper, only the thermal conductivity of FLCC below 100 °C was measured. To simulate and analyze the thermal behavior of buildings made of these types of thermally insulating, lightweight cement composites under fire, it is also needed to obtain the thermal conductivity of FLCC under elevated temperature in future study.

### 3.6. Microstructural Characterization

**Figure 13:**
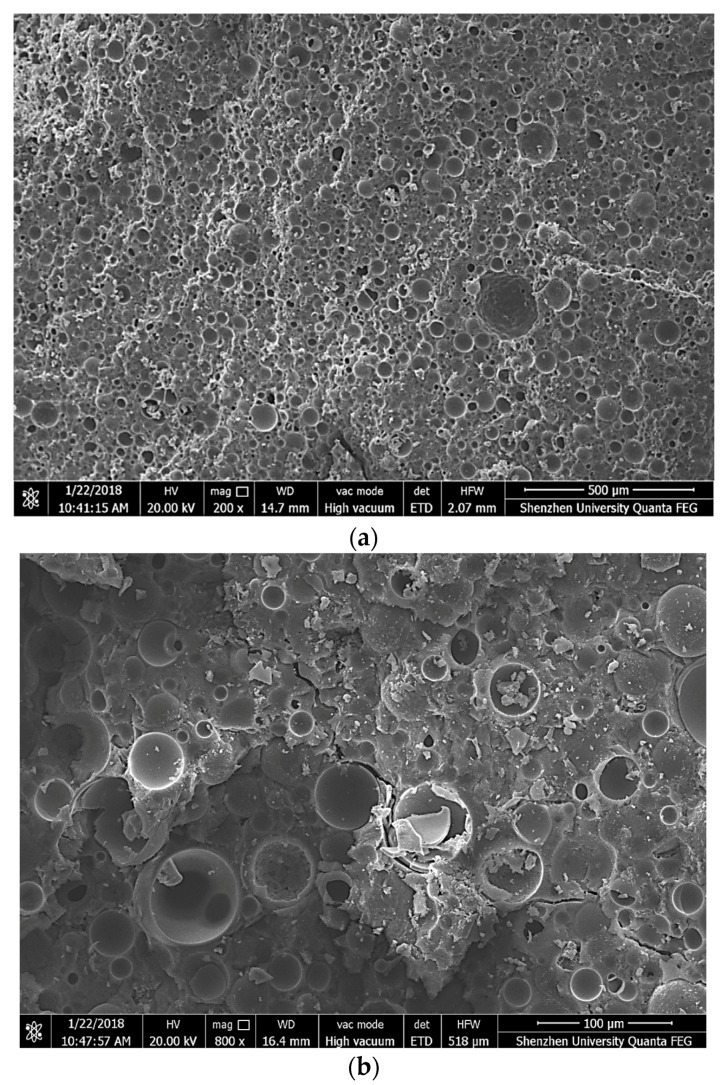
SEM image of fractured surface of FLCC at different magnifications (FL46): (**a**) 500 μm; (**b**) 100 μm.

The morphological and microstructural characterizations of the samples from the damaged concrete segments after the compression test on FLCC cubes were analyzed with a scanning electron microscope (Phenom pro). The test samples were soaked in isopropanol to stop the hydration, and then vacuum-dried in the oven at 40 °C before testing. Figure 13 illustrates the SEM image of FLCC at different magnifications. The microstructure of dense matrix and homogeneous GM can be observed in the composites. In general, the microspheres seemed well distributed in the matrix. Further, the shell of the glass microspheres remained intact even after moist curing for 28 days, indicating that there was little or no interaction between the microspheres and the paste. This means that the glass microspheres can be utilized in cement-based composites. Under stress, the crack initiates from the interfacial transition zone between cement matrix and glass microspheres as shown in Figure 13b. This is contrary to behavior exhibited by lightweight aggregate concrete (LWC), where the crack initiation occurs due to the failure of the aggregates. This figure also shows that glass microspheres offer resistance to crack growth, leading to matrix cracking and debonding prior to GM particle breakage. This is due to the lower strength of the matrix compared to that of GM particles used in this paper that may hinder the crack growth.

## 4. Conclusions

This paper presents a new type of multifunctional, floatable, lightweight cement composite (FLCC) with densities lower than that of water (oven-dry density of 750 kg/m^3^) and compressive strength (**f_cm_**) up to 41 MPa by means of commercially available glass microspheres. A range of properties was investigated for seven different types of FLCC, and FLCC using GM46 was found to have optimal compressive and flexural strength. Addition of 1.5% PE fiber in volume improves the flexural strength by more than 3 times. Under direct tension, FLCC incorporating a low content of PE fiber (1% in volume) exhibits strain-hardening behavior with ultimate tensile strain capacity of 6%. Thus, an ultralightweight FLCC (lightweight ECC) with strain-hardening characteristics has been produced for wider engineering applications. However, the elastic modulus was lower than normal concrete and lightweight concrete due to the lower elastic modulus of the glass microsphere aggregates. The FLCC also exhibited better water absorption capacity and much superior thermal insulation properties. The use of FLCC developed in this paper enables novel metal/polymer-FLCC composite structures to be further developed with lower self-weight, which will provide alternatives for prefabricated concrete structures, marine construction, and ship hulls. This will benefit the transportation and installation of precast and mass-sensitive structures.

## Figures and Tables

**Table 1:** Specification of lightweight concrete in different codes.

Code Practice	Density of Lightweight Concrete *ρ*	28-day Compressive Strength (f_cm_)
CEB-FIP 2010 [6]	Oven-dry: 800–2000 kg/m^3^	LC8-LC80 (8–80 MPa)
ACI 213R-14 [7]	Air-dry: 1440–1850 kg/m^3^	Common value: 21–35 MPa
ASTM C330 [8]	1600–1840 kg/m^3^	Common value: 17–28 MPa
BS EN 13055-2016 [9]	Not exceeding 2000 kg/m^3^	N.A.
JGJ 51-2002 [10]	Oven-dry density ≤ 1950 kg/m^3^	LC5.0-LC60 (10–38.5 MPa)

**Table 2:** Summary of relevant information in literature.

Literature	Lightweight Filler	Density *ρ* (kg/m^3^)	28-day Compressive Strength f_cm_ (MPa)	28-day Flexural Strength f_ct_ (MPa)	Specific Strength (kPa/kgm^−3^)	Elastic Modulus E_c_ (GPa)	Thermal Conductivity λ (W/mK)
Oreshkin et al. [42]	3M^TM^ Glass Bubbles K25	660–993 693–1036	13.9–25.9 15.6–29.0	4.6–5.9 (non-extruded) 5.0–6.6 (extruded)	21.1–26.1 22.5–28.0	–	0.16–0.24 0.15–0.23
Blanco et al. [40]	Cenospheres from coal–burning power plant	1090–1510	5.04–33.03	2.09–5.86	4.62–21.9	–	0.36–0.44
Perfilov et al. [43]	Coated hollow glass microspheres (HGMS)	750–1109	2.2–3.9	0.1–0.8	2.93–3.52	–	–
Korolev et al. [44]	Hollow glass microspheres	1252–1462	45.3–46.5	–	31.9–36.2	–	–
Semenov et al. [45]	Hollow ceramic microspheres	857–957	8.42–18.5	1.98–3.7	9.82–19.3	–	–
Mcbride et al. [54]	Ceramic microspheres	1810–2083	12.1–16.9	0.24–0.33	6.7–8.1	–	–
Hanif et al. [38]	Fly ash cenospheres	1187–1297 (dry:1003–1098)	18.6–23.5	3.7–4.9	15.7–18.1	6.57–8.44	0.31–0.4
Hanif et al. [39]	Fly ash cenospheres	1260–1612	30.38–55.92	5.38–9.29	24.11–34.69	9.18–18.02	–
Hanif et al. [46]	Glass cenospheres	880.2–1375.7	14.3–43.0	4.75–7.89	16.2–31.3	3.68–12.5	-
Hanif et al. [47]	Glass microspheres Fly ash cenospheres	734.8–1564.1 1453.9–1551.3	6.23–47.9 40.4–53.6	0.78–6.7 6.96–11.3	8.48–30.6 27.8–34.6	1.82–15.7 13.1–16.7	-
Al–Gemeel et al. [48]	Spherical 110P8 hollow glass microspheres	2034–2158	55.5–65.0	5.6–14.0	27.3–30.1	-	-
Yang and Li [49]	Hollow glass microspheres 3M^TM^ K20	566.0–1001.0	3.4–5.5	1.06–1.69 (tensile)	6.0–5.49	0.96–1.39	-
Huang et al. [50]	Fly ash cenospheres	1649–1820	25.0–47.6	4.8–5.9 (tensile)	15.2–26.2	-	0.29–0.37
Yun et al. [51]	Fly ash cenospheres	2011–2370	24.6-43.9	1.52–3.16	12.2–18.5	17.6–39.1	1.41–2.21
Wu et al. [26]	Fly ash cenospheres	1154–1471	33.0–69.4	3.6–7.3	28.6–47.2	10.4–17.0	0.28–0.4
Huang et al. [35]	Fly ash cenospheres	1302–1464	46.9–65.8	7.06	36.0–44.9	9.8–13.2	-
Huang et al. [41]	Fly ash cenospheres Hollow glass microspheres	1361 946–969	64. 028.1–32.3	6.7–8. 0-	47. 029.7–33.3	15.4 -	-

**Table 3:** Chemical composition of cement, silica fume, FAC, and GM.

Composition	CaO	SiO_2_	Al_2_O_3_	Fe_2_O_3_	MgO	K_2_O	Na_2_O	B_2_O_3_	SO_3_	C_3_S	C_2_S	C_3_A	C_4_AF
Cement	63.5	19.4	4.8	2.8	1.3	0.4	0.2	-	1.9	69.0	3.6	8.1	8.4
Silica fume	0.2	94.1	0.6	0.1	0.4	0.3	0.1	-	N.A	N.A.	N.A.	N.A.	N.A.
FAC	-	51.6	34.6	-	0.7	-	-	-	-	-	-	-	-
GM	13.4	72.7	1.0	0.0	0.0	0.0	6.2	6.4	-	-	-	-	-

**Table 4:** Specification of FAC and GM.

Type	Bulk Density (g/cm^3^)	Crushing Strength (MPa)	Moisture Content (%)	Flotation Ratio (%)
FAC	0.45	15.0	0.2	95
GM40	0.38–0.42	27.6	0.5	92
GM46	0.44–0.48	41.3	0.5	92
GM60	0.57–0.63	68.9	0.5	92

**Table 5:** Mix proportions of FLCC.

Mix ID	Fillers	Mix Proportion of Matrix by Volume of Total Binder					
		Water/Binder	B ^1^		Fillers/B	SP ^6^/B	Fiber (V%)
			C ^2^	SF ^3^			
ULCC-1 [26]	FAC ^4^	0.35	0.92	0.08	0.38	0.01	-
FL40	GM40 ^5^	0.60	0.85	0.15	0.56	0.02	-
FL46	GM46	0.60	0.85	0.15	0.59	0.02	-
FL60	GM60	0.60	0.85	0.15	0.66	0.02	-
FL46-35	GM46	0.35	0.85	0.15	0.59	0.05	-
FL46PE05	GM46	0.65	0.85	0.15	0.59	0.02	0.5
FL46PE10	GM46	0.65	0.85	0.15	0.59	0.04	1.0
FL46PE15	GM46	0.65	0.85	0.15	0.59	0.05	1.5
LWC ^7^	Expanded shale	0.35	0.90	0.1	0.6	0.02	-
NWC ^8^	Granite	0.45	1	0	-	0.01	-

^1^ B = binder; ^2^ C = cement; ^3^ SF = silica fume; ^4^ FAC = fly ash cenospheres; ^5^ GM = glass microspheres; ^6^ SP = superplasticizer; ^7^ LWC is lightweight concrete using expanded shale aggregates while ^8^ NWC is normal weight concrete using granite aggregates, both with 28-day compressive strength similar to ULCC.

**Table 6:** Test method references and specimen sizes.

Property	Test Standard	Testing Age	Specimen Type and Size (mm)	No. of Tests
Workability (flow table)	BS EN 1015-3 [55]	Right after mixing	-	-
Estimated porosity	ASTM C 138 [56]	-	-	-
Density of hardened specimens after demold	ASTM C 138 [56]	1–2 days	Cube:50 × 50 × 50	3
Compressive strength	ASTM C109 [58] ASTM C349 [62] BS EN 196-1 [61] ASTM C39 [57]	28 days	Cube:50 × 50 × 50 Cube:40 × 40 × 40 - Cylinder:100 × 200	3 2 - 3
Flexural strength	BS EN 196-1 [61]	28 days	Prism: 40 × 40 × 160	3
Tensile strength	JSCE-2008 [63]	28 days	Dog-bone specimens	3
Elastic modulus Poisson’s Ratio	ASTM C469 [59]	28 days	Cylinder:100 × 200	3
Thermal conductivity	ASTM C518 [64]	Around 100 days ^1^	Slab: 300 × 300 × 30	2
Water sorptivity test	ASTM C 1585 [66]	28 days	Cylinder: 100 × 50	3

^1^ 28 days moist curing followed by drying in lab air for 14 days then in oven at 105 °C to constant weight.

**Table 7:** Measured properties of FLCC.

Type	Flow/Sump (mm)	Fresh Density *ρ* (kg/m^3^)	28-day Compressive Strength f_cm_ (MPa)	28-day Flexural Strength f_ct_ (MPa)	E_c_ (GPa)	*v*_c_	Estimated Porosity (%)	Specific Strength (kPa/kgm^−3^)
ULCC-1 [26]	200	1471	69.4	7.3	17.0	0.25	6.6	47.2
FL40	160	970	41.0 (2.8)	3.5(0.4)	-	-	1.2	42.3
FL46	164	940	39.5 (1.9)	3.2(0.4)	7.2	0.25	1.4	42.0
FL60	160	890	31.1 (0.5)	2.2(0.9)	-	-	6.4	34.9
FL46-35	120	880	32.1 (1.7)	2.7(0.2)	-	-	7.5	36.5
FL46PE05	155	940	26.9 (2.4)	4.0(0.7)	-	-	0.9	28.6
FL46PE10 ^1^	140	940	27.3 (1.9)	7.2(0.4)	-	-	1.2	29.0
FL46PE15	134	930	31.0 (3.3)	9.5(0.5)	-	-	1.7	33.3
LWC	100 (slump)	1850	53.2	-	-	0.25	2.4	28.7
NWC	105 (slump)	2360	68.0	-	-	-	8.8	28.8

^1^ FL46PE10 stands for FLCC using GM46 with 1% PE fiber in volume; E_c_ = Elastic Modulus; *v*_c_ = Poisson’s ratio; LWC = lightweight concrete using expanded shale aggregate. The value in the bracket represents the standard deviation.

**Table 8:** Density, mechanical properties, and thermal conductivity.

Mix ID	w/b	Aggregate Type	1-Day Density *ρ*_1_ (kg/m^3^)	Oven-Dry Density *ρ_o_* (kg/m^3^)	28-Day Compressive Strength f_cm_ (MPa)	Thermal Conductivity λ (W/mK)
FL46 ^1^	0.60	GM	940	750	39.5	0.154 (10 °C) 0.162 (30 °C) 0.180 (90 °C)
ULCC-1 [26]	0.35	FAC	1471	1303	69.4	0.40
ULCC-6 [26]	0.56	FAC	1154	966	33.0	0.28
Concrete [26]	0.42	Granite	2341	2251	67.6	1.98

^1^ For FL46, the thermal conductivity is the average value of two specimens.
